# Discovery and Characterization of a Novel MASTL Inhibitor MKI-2 Targeting MASTL-PP2A in Breast Cancer Cells and Oocytes

**DOI:** 10.3390/ph14070647

**Published:** 2021-07-05

**Authors:** Minsung Kang, Chijung Kim, Jiyeon Leem, Ye-hyun Kim, Young-ju Kwon, Yi Na Yoon, Chong Hak Chae, Jiyeon Ahn, Kwan-Young Jung, Jeong Su Oh, Jae-Sung Kim

**Affiliations:** 1 Division of Radiation Biomedical Research, Korea Institute of Radiological and Medical Sciences, Seoul 139-706, Korea; mkang@kirams.re.kr (M.K.); ye4978@gmail.com (Y.-h.K.); youngju@kirams.re.kr (Y.-j.K.); yinay@kirams.re.kr (Y.N.Y.); ahnjy@kirams.re.kr (J.A.); 2 Therapeutics & Biotechnology Division, Korea Research Institute of Chemical Technology, Daejeon 34114, Korea; rlacl93@krict.re.kr (C.K.); chchae@krict.re.kr (C.H.C.); 3 Department of Integrative Biotechnology, Sungkyunkwan University, Suwon 16419, Korea; limjiyeon0619@gmail.com; 4 Radiological and Medico-Oncological Sciences, University of Science and Technology, Daejeon 34113, Korea; 5 Department of Medicinal Chemistry and Pharmacology, University of Science and Technology, Daejeon 34113, Korea

**Keywords:** MASTL, MASTL inhibitor, PP2A, antitumor activity, breast cancer

## Abstract

Although microtubule-associated serine/threonine kinase-like (MASTL) is a promising target for selective anticancer treatment, MASTL inhibitors with nano range potency and antitumor efficacy have not been reported. Here, we report a novel potent and selective MASTL inhibitor MASTL kinase inhibitor-2 (MKI-2) identified in silico through a drug discovery program. Our data showed that MKI-2 inhibited recombinant MASTL activity and cellular MASTL activity with IC_50_ values of 37.44 nM and 142.7 nM, respectively, in breast cancer cells. In addition, MKI-2 inhibited MASTL kinase rather than other AGC kinases, such as ROCK1, AKT1, PKACα, and p70S6K. Furthermore, MKI-2 exerted various antitumor activities by inducing mitotic catastrophe resulting from the modulation of the MASTL-PP2A axis in breast cancer cells. The MKI-2 treatment showed phenocopies with MASTL-null oocyte in mouse oocytes, which were used as a model to validate MKI-2 activity. Therefore, our study provided a new potent and selective MASTL inhibitor MKI-2 targeting the oncogenic MAST-PP2A axis in breast cancer cells.

## 1. Introduction

Microtubule-associated serine/threonine kinase-like (MASTL), also known as Greatwall kinase, is a key mitotic kinase that regulates mitotic progression and maintains mitotic integrity [1,2,3]. MASTL controls the inactivation of the protein phosphatase 2A complex (PP2A-B55) by the directly phosphorylating its substrates, such as α-endosulfine (ENSA) and cAMP-regulated phosphoprotein 19, during mitosis and meiosis in mammalian somatic cells and oocytes, respectively [4,5,6,7,8,9]. PP2A is the main phosphatase that dephosphorylates various mitotic regulatory proteins during mitosis and meiosis [4,10]. Therefore, the MASTL-PP2A axis is a key regulator of the M-phase-promoting factor (MPF) during mitosis and meiosis, particularly in highly proliferative cells [4].

Recent studies suggested that MASTL acts as an oncogenic kinase by inactivating tumor suppressive PP2A-B55 complex in breast cancer cells [11,12,13], thereby regulating various oncogenic properties, such as cellular transformation, chromosome instability, and metastasis [3,14,15]. MASTL targeting has reduced tumor growth in various in vitro and in vivo tumor models [13,16]. Notably, MASTL are highly expressed in multiple types of cancers, including breast, head and neck, gastric, thyroid, and colorectal cancers [12,15,17,18,19,20]. MASTL inhibition selectively eradicated proliferative cancer cells rather than normal cells by inducing mitotic catastrophe [12,21,22]. Therefore, accumulating evidence clearly indicates that MASTL is an attractive, druggable target for selective anticancer treatment.

Recently, several groups reported candidate compounds as potential MASTL inhibitors [22,23,24]. Greatwall kinase inhibitor-1 (GKI-1) was reported as a first-line inhibitor against MASTL, which was designed by using the solved protein structure of the kinase domain of MASTL [23]. GKI-1 can inhibit MASTL in vitro and disrupt mitotic events by decreasing phosphorylated ENSA with μM range potency in HeLa cells [23]. However, GKI-1 did not show the anticancer activity in breast cancer cells [22]. Further, two compounds identified in silico in another study could be potential MASTL inhibitors without any validated biochemical data [24]. We previously reported MASTL kinase inhibitor-1 (MKI-1), a new MASTL inhibitor, showing antitumor activities across in vitro and in vivo tumor models by inhibiting MASTL in breast cancer cells [22]. Nonetheless, MKI-1 also showed μM range potency and efficacy for MASTL inhibition [22]. In the present study, we developed a second-generation MASTL inhibitor MKI-2 using an in silico-based drug discovery program involving a docking model. MKI-2 showed nM range potency and efficacy for MASTL inhibition in breast cancer cells. In addition, we demonstrated that MKI-2 treatment showed a phenocopy with MASTL-null oocyte in mouse oocytes, which were used as a model to validate MKI-2 activity. Thus, our data provided a new potent MASTL inhibitor MKI-2.

## 2. Results

### 2.1. Identification and Validation of MASTL Inhibitor MKI-2 In Silico and In Vitro

**Figure 1:**
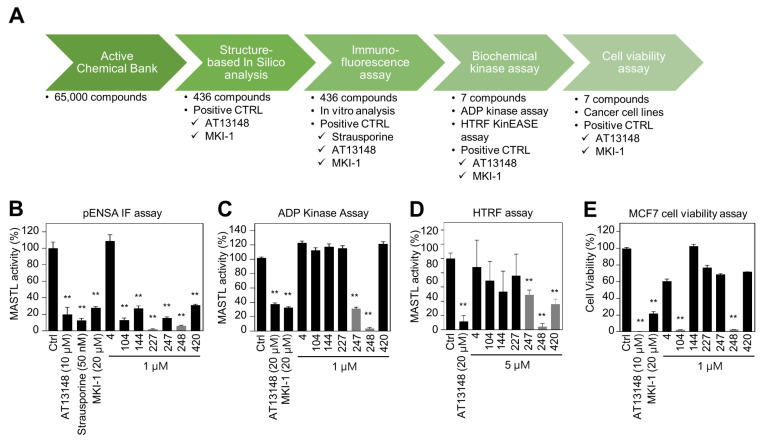
Identification of MASTL inhibitor MKI-2 using in silico and in vitro analyses (**A**) Schematic summary of in silico-based drug discovery process. Immunofluorescence (**B**), the ADP-Glo^TM^ kinase assay (**C**) and HTRF kinase assay (**D**) were performed to determine MASTL activities under the treatments of two positive controls and candidate compounds. (**E**) MCF7 cells were treated with DMSO (Ctrl), 20 µM AT13148, 20 µM MKI-1, and 1 µM candidate compounds for 72 h. Cell viability was measured using the WST-8 assay. The data are presented as the mean ± standard deviation of three independent experiments. ** *p* < 0.01.

**Figure 2:**
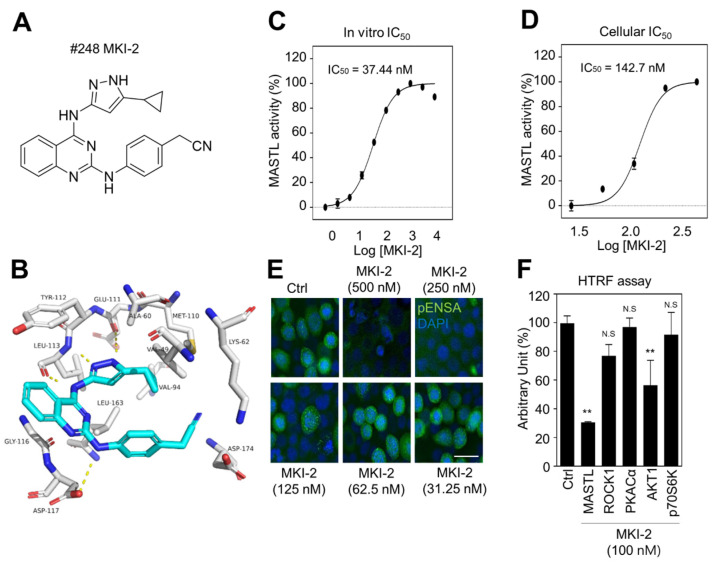
Validation of MASTL inhibitor MKI-2 using in silico and in vitro analyses (**A**) The chemical structure of MKI-2. (**B**) The docking model of MKI-2 and the MASTL kinase domain. The in vitro or cellular IC_50_ of MKI-2 was determined by in vitro kinase assay (**C**) and the IN Cell Analyzer HCA System (**D**). In at least 4 images, each image that contains more than 500 cells was analyzed from each group by the IN Cell analyzer. (**D**,**E**) Immunofluorescence staining was performed using anti-phospho(Ser67) ENSA (green) in MCF7 cells after arrested in mitosis using colcemide (80 ng/mL) and various concentrations of MKI-2 for 14 h. Scale bars = 20 µm. (**F**) HTRF kinase assay were performed using recombinant AGC kinases to determine AGC kinases activities with 100 nM MKI-2. The data are presented as the mean ± standard deviation of three independent experiments. ** *p* < 0.01. N.S.: not significant.

Since MKI-1, a first-line inhibitor of MASTL, showed µM range potency for the inhibition of MASTL [22], more potent and selective MASTL inhibitors were further screened in an in silico-based drug discovery program using a docking model for the MASTL-MKI-1 interaction. Briefly, 436 compounds were selected by the structure-based in silico analysis from 65,000 active chemical library. A total of 436 compounds were further analyzed using various biochemical assays, including the IN-Cell-based immunofluorescence, homogeneous time-resolved fluorescence (HTRF) KinEASE, ADP kinase, and cell viability assay (Figure 1A). In these experiments, AT13148, a multiple-AGC kinase inhibitor [25], and MKI-1 were used as the positive controls. As a result, seven compounds were selected by the biochemical screens. As shown in Figure 1B–E, #248 (2-(4-((4-((5-cyclopropyl-1*H*-pyrazol-3-yl)amino)quinazolin-2-yl)amino)phenyl)acetonitrile) compound was finally selected as a potential candidate for the MASTL inhibitor because of the highest potency for the inhibition of MASTL. Thus, a #248 compound, named MKI-2, emerged as a new candidate compound for inhibiting MASTL (Figure 2A). The two important amino acid residues, LEU-113 and GLU-111, was predicted to interact with the pyrazole group of MKI-2 through strong hydrogen bonding. The terminal cyclopropyl moiety attached to the pyrazole group interact with hydrophobic interaction with VAL-94. These hydrogen bonding and hydrophobic interactions allowed MKI-2 to face the correct position for entry into the binding pocket of the MASTL active site. The “hinge” region of the MASTL existed between ASP-117 and LEU-163 residues, allowing other strong interactions between aminoquinazolin scaffold and ASP-117 key residue. The docking model predicted that the phenylacetonitrile moiety of MKI-2 is faced to the solvent explosion region (Figure 2B). In vitro kinase assay using recombinant MASTL indicated that in vitro IC_50_ of MKI-2 was 37.44 nM (Figure 2C). The cellular IC_50_ was 142.7 nM, as determined by a dose-response curve analysis of MKI-2 using immunofluorescence analysis for phospho-ENSA, a cellular substrate of MASTL (Figure 2D,E), suggesting that MKI-2 is a potent MASTL inhibitor with nM range potency. We further tested whether MKI-2 inhibited other four AGC kinases, including ROCK1, AKT1, PKA Cα, and p70S6K, since AGC kinases have a similar ATP-binding pocket in the kinase domain [26]. Notably, the in vitro kinase assay indicated that MKI-1 more selectively inhibited MASTL than the other AGC kinases (Figure 2F). Collectively, our data indicated that MKI-2 is a potent and selective MASTL inhibitor with nM range potency.

### 2.2. MKI-2 Induces Mitotic Catastrophe of Breast Cancer Cells via MASTL-PP2A

**Figure 3:**
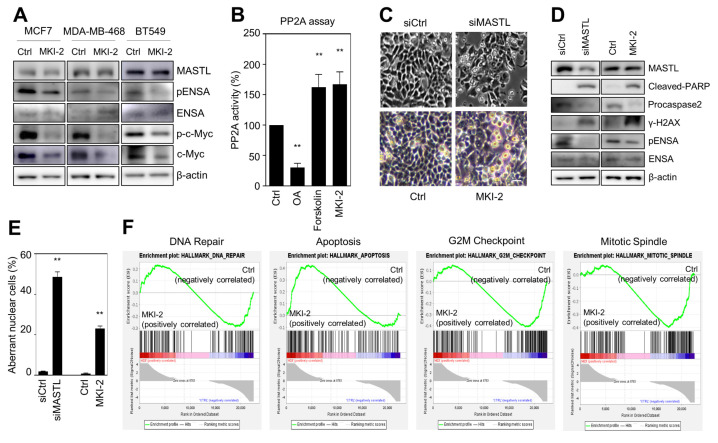
MKI-2 induces mitotic catastrophe of breast cancer cells via MASTL-PP2A regulation (**A**) MCF7, BT-549, and MDA-MB-468 cells were treated with DMSO (Ctrl) or 250 nM of MKI-2 for 12 h. The cell lysates were analyzed by immunoblotting with the indicated antibodies. β-actin was used as the loading control. (**B**) PP2A activity in MCF7 cells was measured after treatment of DMSO (Ctrl), 50 nM OA, 160 µM forskolin, or 250 nM MKI-2 for 24 h. (**C**–**F**) MCF7 cells were transfected with control siRNA or MASTL siRNA, or treated with DMSO(Ctrl) or 250 nM MKI-2 for 24 h. (**C**) The representative cell images. Scale bars = 20 µm. (**D**) The cell lysates were analyzed by immunoblotting with the indicated antibodies. β-actin was used as the loading control. (**E**) The aberrant nuclear cells, such as nuclei with irregular shapes or fragmented nuclei were scanned and determined using the IN Cell Analyzer HCA System. In at least 17 images, each image that contains more than 100 cells was analyzed from each group by the IN Cell analyzer. (**G**) mRNA Transcripts from MKI-2-treated cells were analyzed by GSEA software. The data represent typical results and are presented as the mean ± standard deviation of three independent experiments. ** *p* < 0.01.

To validate that MKI-2 inhibited MASTL in breast cancer cells, we first examined whether MKI-2 inhibits phospho-ENSA in three breast cancer cell lines, including MCF7, BT549, and MDA-MB468 cells. Our data indicated that MKI-2 inhibited phosphorylated-ENSA in the three breast cancer cell lines (Figure 3A). In addition, we examined whether MKI-2 increases PP2A activity and inhibits c-Myc since we previously observed that MASTL inhibition activates PP2A and decreases c-Myc stability in breast cancer cells [12,22]. Forskolin and OA were used as positive and negative controls in the PP2A activity assay, respectively. Our data indicated that MKI-2 can increase PP2A activity and decrease c-Myc proteins (Figure 3A,B), suggesting that MKI-2 inhibits the MASTL-PP2A-c-Myc axis in breast cancer cells.

Since our previous reports showed that MASTL depletion causes mitotic catastrophe of breast cancer cells [12,22], we next evaluated whether MKI-2 induces mitotic catastrophe. Notably, the microscopic analysis indicated that MKI-2 induced the mitotic arrest of MCF7 cells, which was similar to the mitotic arrest phenotype in MASTL-depleted MCF7 cells (Figure 3C). In addition, we observed increased levels of cleaved-PARP and γ-H2AX and decreased procaspase-2 and phospho-ENSA in MKI-2-treated MCF7 cells, which was consistent with the result in the MASTL-depleted MCF7 cells (Figure 3D). Furthermore, MKI-2 increased aberrance in nuclei of MCF7 cells (Figure 3E), consistent with the observation that MASTL depletion or inhibition increased aberrant nuclei, such as nuclei with irregular shapes or fragmented nuclei in MCF7 cells [12,22]. Moreover, the gene set enrichment (GSEA) analysis from transcript analysis in MKI-2-treated MCF7 cells showed that MKI-2 modulated G2/M arrest, apoptosis, mitotic spindle, and DNA repair of MCF7 cells (Figure 3F), which are the main functions of MASTL [13]. Taken together, our results suggested that MKI-2 causes mitotic catastrophe in breast cancer cells by modulating the MASTL-PP2A axis.

### 2.3. MKI-2 Inhibits the Oncogenic Properties and Enhances the Radiosensitivity of Breast Cancer Cells

**Figure 4:**
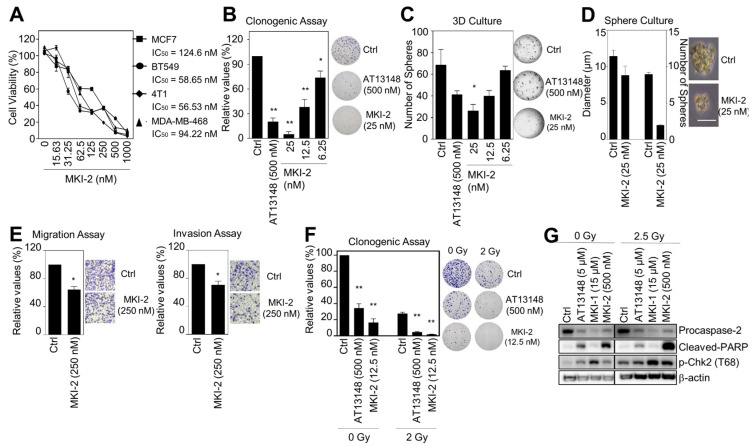
MKI-2 inhibits the oncogenic properties and enhances the radiosensitivity of breast cancer cells (**A**) MCF7, BT549, 4T1, and MDA-MB-468 cells were treated with serial dilutions of MKI-2 from 1000 nM for 72 h. The cell viabilities were measured using the WST-8 assay. (**B**–**E**) MCF7 cells were treated with DMSO (Ctrl), 500 nM AT13148, and the indicated concentrations of MKI-2. (**B**) Colony formation was determined using the colony formation assay. Representative images (right panel) of colony formation of MCF7 cells. (**C**) Spheroid formation was determined using the 3D culture assay. (**D**) Mammosphere formation was determined using the sphere formation assay. Scale bars = 100 μm. Representative images (right panel) of the colony, spheroid, and tumorsphere formation of MCF7 cells (**B**–**D**). (**E**) BT549 cells were used for migration and invasion assays. Representative images (right panel) of migration and invasion of BT549 cells. (**F**,**G**) MCF7 cells were treated with DMSO (Ctrl), 0.5 μM AT13148, and 12.5 nM MKI-2 with or without 2.5 Gy radiation for 14 days (**F**) or 24 h (**G**). Colony formation was determined by using the colony formation assay. Representative images (right panel) of the colony formation of MCF7 cells treated with indicated conditions. Cell lysates were analyzed by immunoblotting with the indicated antibodies (**G**). The data represent typical results and are presented as the mean ± standard deviation of three independent experiments. * *p* < 0.05, ** *p* < 0.01.

MASTL inhibition reduces the oncogenic properties and enhanced the radiosensitivity of breast cancer cells [12]. Thus, we next examined the antitumor activities of MKI-2 in breast cancer cells through various analyses, including cell viability, clonogenic, mammosphere formation, 3D culture, and invasion and migration assays. The results indicated that MKI-2 inhibited the proliferation of various breast cancer cells, including the human breast cancer MCF7, BT549, and MDA-MB468 cells and mouse breast cancer 4T1 cells with nM range efficacy (IC_50_ of MKI-2 was 56~124 nM, dependent on cancer cell lines) (Figure 4A). MKI-2 also clearly inhibited the colony and mammosphere formation of MCF7 cells with nM range efficacy (Figure 4B–D). Furthermore, MKI-2 inhibited the migration and invasion of BT549 cells (Figure 4E), indicating that MKI-2 can inhibit the oncogenic properties of breast cancer cells. Next, we found that MKI-2 decreased colony formation in MCF7 cells in response to irradiation (Figure 4F), with increased cleaved-PARP and phospho-Chk2 and decreased procaspase-2 (Figure 4G), which was more potent than AT13148 and MKI-1, suggesting that MKI-2 can enhance the radiosensitivity of breast cancer cells. Therefore, these results collectively suggested that MKI-2 has potent antitumor activities in breast cancer cells.

### 2.4. MKI-2 Treatment Phenocopies the MASTL-Null Oocytes

**Figure 5:**
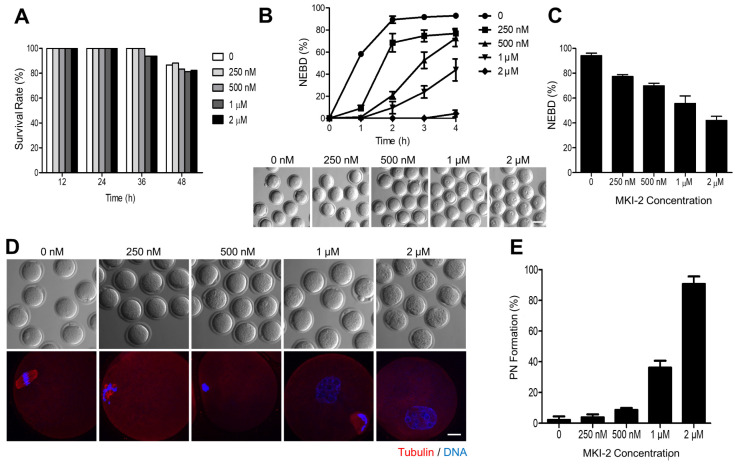
Validation of MKI-2 activity using mouse oocytes (**A**) GV oocytes were treated with 0, 0.25, 0.5, 1.0, and 2.0 μM MKI-2 and scored for survival rate up to 48 h. (**B**) After 30 min of culture with MKI-2 and IBMX, GV oocytes were released from prophase arrest by washing out IBMX and were scored for GVBD. The percentage of GVBD was expressed as mean ± SEM from three independent experiments. Representative images at 4 h of culture following IBMX release were shown. Scale bar, 100 μm. (**C**) After 18 h of culture with MKI-2 in the absence of without IBMX, the percentage of GVBD was scored and expressed as mean ± SEM from three independent experiments. (**D**) MII oocytes were collected and treated with MKI-2 in M2 medium for 8 h. Oocytes were fixed and stained with anti-tubulin antibodies. DNA was counterstained with DAPI. Scale bar, 20 μm. (**E**) Nuclei with decondensed chromatin were evaluated. Data were expressed as mean ± standard deviation of three independent experiments.

As the function of MASTL has been well characterized in oocytes [8,9,27,28], we validated the MKI-2 activity using mouse oocytes by comparing phenotypes. First, we treated immature oocytes with MKI-2 and examined the cytotoxicity. The survival rate of oocytes treated with various concentrations of MKI-2 was not significantly different among groups (Figure 5A), suggesting that MKI-2 is not toxic up to oocytes. We then examined the effect of MKI-2 on meiotic cell cycle. Consistent with recent studies that the activation of MASTL is essential for G2-M transition [5,29], MKI-2 treatment effectively inhibited GVBD (indicative of the entry into the meiotic M phase in oocytes) in a dose-dependent manner. Moreover, most oocytes remained in the GV stage even after prolonged culture (Figure 5B,C), implying that the effect of MKI-2 is not transient in oocytes. It has been recently shown that MASTL-null oocytes reassembled a nuclear structure containing decondensed chromatin [28]. Therefore, we investigated whether MKI-2 could induce pronuclear formation in oocytes. Notably, oocytes formed distinct nuclei with decondensed chromatin after MKI-2 treatment (Figure 5D,E). Taken together, our results reveal that treating oocytes with MKI-2 displayed similar phenotypes, observed as MASTL-depleted or -null oocytes, corroborating that MKI-2 has the capacity to inhibit MASTL.

## 3. Discussion

Although recent studies suggest that MASTL is a promising target for selective anticancer treatment [16], MASTL inhibitors with nM range potency and antitumor efficacy have not been reported. Our previous report showed that MKI-1 was an MASTL inhibitor with antitumor activities in breast cancer cells. However, it showed relatively low potency for MASTL inhibition with IC_50_ of 9.9 μM. In the current study, our data reported the second-generation MASTL inhibitor MKI-2, a new potent MASTL inhibitor with in vitro IC_50_ of 37.44 nM and cellular IC_50_ of 142.7 nM in breast cancer cells. In addition, MKI-2 did not modulate other AGC kinases. Furthermore, we found that MKI-2 had potent antitumor activities by disrupting mitosis via the MASTL-PP2A-Myc regulation in breast cancer cells. Moreover, MKI-2 treatment inhibited GVBD and formed distinct nuclei with condensed chromatin in mouse oocytes, which phenotypes were a clear phenocopy with MASTL-null oocytes [28]. Therefore, this study presents a new potent and selective MASTL inhibitor MKI-2 targeting MASTL-PP2A in breast cancer cells.

Two compounds, GKI-1 and MKI-1, were recently reported as MASTL inhibitors [22,23]. GKI-1 inhibited MASTL with in vitro IC_50_ of 10 μM and reduced phosphorylated ENSA with mitotic disruption in HeLa cells [23]. However, it showed no anticancer activities in breast cancer cells [22]. Our previous report also showed that MKI-1 inhibited MASTL with in vitro IC_50_ of 9.9 μM and reduced phosphorylated ENSA with the induction of aberrant nuclear cells in breast cancer cells [22]. Compared with GKI-1, MKI-1 showed anticancer activities in in vitro and in vivo breast cancer models [22]. However, the in vitro IC_50_ of both GKI-1 and MKI-1 for the inhibition of MASTL was approximately 10-fold less than that of the multiple AGC kinase inhibitor AT13148 [22], indicating that both compounds are ineffective as MASTL inhibitors. Compared with GKI-1 and MKI-1, MKI-2 showed the superior in vitro inhibition of MASTL (37.44 nM of in vitro IC_50_ and 142.7 nM of cellular IC_50_). In addition, MKI-2 can induce the mitotic arrest phenotype of breast cancer cells, which were identical to the phenotype of MASTL-depleted breast cancer cells. Moreover, MKI-2 treatment showed the phenocopy of MASTL-null oocytes in mouse oocytes. Furthermore, MKI-2 did not modulate other AGC kinases, including ROCK1, AKT, p70S6K, and PKA Cα. Therefore, our data suggested that MKI-2 is a new, potent, and selective MASTL inhibitor.

Our data showed that MKI-2 had superior anticancer activities with nM range efficacy in breast cancer cells. MKI-2 showed approximately 50-fold more potent anticancer activity than that of AT13148 in clonogenic and 3D culture analysis of breast cancer cells. In addition, MKI-2 can also enhance the radiosensitivity of breast cancer cells with nM range efficacy, indicating the superior anticancer activity of MKI-2. In addition, we also observed that MKI-2 disrupted mitosis of breast cancer cells by modulating MASTL-PP2A-c-Myc regulation, a mechanism which is consistent with the results in the MASTL-depleted cells [12,22]. Thus, our data suggested that MKI-2 demonstrates anticancer activity in breast cancer cells by regulating MASTL-PP2A during mitosis.

## 4. Materials and Methods

### 4.1. Cell Culture

Cell lines were purchased from American Type Culture Collection (ATCC; Manassas, VA, USA). MCF7, 4T1, and MDA-MB-468 cells were maintained in DMEM (Corning, NY, USA) supplemented with 10% fetal bovine serum (FBS; Corning Inc., Corning, NY, USA) and 1% penicillin/streptomycin. BT549 cells were cultured in RPMI 1640 (Welgene, Daegu, Korea) containing 10% FBS and 1% penicillin/streptomycin. MCF10A cells were cultured in DMEM/F12 (Invitrogen, Waltham, MA, USA) supplemented with 5% heat-inactivated horse serum (Invitrogen), 1% penicillin/streptomycin, 20 ng/mL EGF (Peprotech, London, UK), 0.5 mg/mL hydrocortisone (Sigma-Aldrich, St. Louis, MO, USA), 100 ng/mL cholera toxin (Sigma-Aldrich), and 10 μg insulin (Sigma-Aldrich). Cells were maintained in a standard condition of 5% CO_2_ at 37 °C.

### 4.2. In Silico Screening

In silico screening from the chemical library that included 65,000 bio-active compounds (Korea Chemical Bank of Korea Research Institute of Chemical Technology (http://www.chembank.org (accessed on 14 November 2021); Daejeon, Korea) was performed as previously described [22].

### 4.3. Chemicals and Treatments

AT13148 was purchased from Selleck Chemicals (Houston, TX, USA) and GKI-1, MKI-1, and MKI-2 synthesized by Korea Research Institute of Chemical Technology (Daejeon, Korea) were treated at the indicated concentrations for each experiment.

### 4.4. Synthesis of MKI-2

#### 4.4.1. 2-Chloro-*N*-(5-cyclopropyl-1*H*-pyrazol-3-yl)quinazolin-4-amine (compound 3)

Briefly, 5-Cyclopropyl-1*H*-pyrazol-3-amine (300 mg, 2.44 mmol) and 2,4-dichloroquinazoline (533 mg, 2.68 mmol) were dissolved in anhydrous acetonitrile (30 mL) followed by the addition of triethylamine (680 µL, 4.88 mmol). The resulting solution was stirred for 12 h at 30 ℃. After the starting material (compound 2) was converted to the new spot through TLC, the solvent was removed by evaporation. The white precipitate was obtained by filtration and dried to obtain the desired compound 3 (558 mg, 80 %). The following data were obtained: 1H-NMR (400 MHz, DMSO-d6) δ 12.29 (bs, 1H), 10.72 (s, 1H), 8.56 (d, 1H, J = 7.8 Hz), 7.77 (dd, 1H, J = 7.8Hz, 7.2 Hz), 7.65 (d, 1H, J = 7.8 Hz), 7.52 (dd, 1H, J = 7.8 Hz, 6.8 Hz), 3.30 (bs, 1H), 1.92 (m, 1H), 0.91 (m, 2H), 0.69 (m, 2H); 13C-NMR (100 MHz, DMSO-d6) δ 159.1, 156.9, 151.3, 147.0, 146.1, 134.2, 127.2, 127.1, 124.0, 113.8, 96.2, 8.4, 7.6; LCMS (ESI+) *m/z* calculated for C14H12ClN5 ([M+H+]) 286.0, found 285.9.

#### 4.4.2. 2-(4-((4-((5-Cyclopropyl-1*H*-pyrazol-3-yl)amino)quinazolin-2-yl)amino)phenyl)acetonitrile (MKI-2)

Compound 3 (500 mg, 1.75 mmol) and 4-aminobenzyl cyanide (254 mg, 1.93 mmol) were dissolved in tert-butanol (25 mL) followed by the addition of concentrated HCl (200 µL). The resulting solution was stirred for 5 h at 100 ℃. After the starting material (compound 3) was converted to the new spot through TLC, the pale white precipitate was collected by filtration washed with tert-BuOH and dried under vacuum to afford the desired product MKI-2 (553 mg, 83%). The following data were obtained: 1H-NMR (400 MHz, DMSO-d6) δ 11.42 (s, 1H), 10.81 (s, 1H), 8.62 (d, 1H, J = 7.8 Hz), 7.82 (dd, 1H, J = 7.8 Hz, 7.2 Hz), 7.56 (d, 1H, J = 8.0 Hz), 7.52 (d, 2H, J = 8.0 Hz), 7.44 (dd, 1H, J = 7.8 Hz, 7.2 Hz), 7.38 (d, 2H, J = 8.0 Hz), 6.11 (bs, 1H), 4.06 (s, 2H), 1.81 (m, 1H), 0.93 (m, 2H), 0.54 (m, 2H); 1H-NMR (100 MHz, DMSO-d6) δ 160.0, 159.3, 158.9, 152.6, 147.0, 146.1, 136.5, 136.0, 129.1, 125.5, 125.2, 125.0, 120.0, 118.2, 111.0, 96.2, 22.7, 8.5, 7.6; LCMS (ESI+) *m/z* calc’d for C22H19N7 ([M+H+]) 381.0, found 381.2.

### 4.5. Immunofluorescence

Immunofluorescence analysis was performed as previously described [30,31]. Briefly, the cells were fixed with 4% paraformaldehyde, permeabilized, and blocked with 0.1 Triton X-100 and 5% fetal calf serum in PBS. The fixed cells were consecutively incubated with primary antibodies against phospho-ENSA (Ser67)/ARPP19 (Ser62) (Cell Signaling Technology; 1:100), and secondary antibodies, such as anti-mouse Alexa-488 and anti-rabbit Alexa-594 (Molecular Probes, Eugene, OR, USA; 1:200). The slides were mounted in DAPI-containing medium and images of the bands were then obtained by using IN Cell analyzer 2000 (GE Healthcare, Chicago, IL, USA).

### 4.6. HTRF Assay

The recombinant GST-tagged-MASTL (Thermo Fisher Scientific) was used for a kinase assay based on HTRF. An HTRF KinEASE assay kit (CisBio, Bedford, MA, USA) was used according to the manufacturer’s instructions.

### 4.7. In Vitro Kinase Assay

Two recombinant GST-tagged-MASTL (Thermo Fisher Scientific) and His-tagged-ENSA (Sino Biological Inc., Beijing, China) were employed for the in vitro kinase assays. The in vitro kinase assay was performed as previously described [22].

### 4.8. Cell Viability Assays

Breast cancer cell lines, including MCF7, BT549, 4T1, and MDA-MB-468 cells, were treated with serial dilutions of MKI-2 from 1 µM to 15.63 nM for 72 h. Cell viability was measured by WST-8 assay (Cyto X^TM^ cell viability assay kit; LPS solution, Daejeon, Korea) according to the manufacturer’s instructions.

### 4.9. RNA Interference

Non-silencing siRNA, a negative control, and MASTL siRNA (5′-GAAUGAACUUGCAUAAUUAUU-3′) were synthesized and purchased from Bioneer (Deajeon, Korea). G-fectin (Genolution, Seoul, Korea) was used for transfection of siRNAs following the manufacturer’s instructions [12].

### 4.10. Clonogenic and Sphere Formation Assay

A clonogenic assay [32] and sphere formation assay [12,33] were performed as previously described.

### 4.11. Three-Dimensional Culture

A 3D culture was performed as recommended by the manufacturer (TheWell Bioscience, North Brunswick Township, NJ, USA).

### 4.12. Invasion and Migration Assay

The invasion assay was performed using a Transwell chamber (Corning Inc.). The inserting chamber was coated with 0.5 mg/mL Matrigel for 2 h at 37 °C, and BT549 cells (5 × 10^4^) were treated with DMSO or 250 nM MKI-2. The lower chamber was filled with RPMI containing 10% FBS, and the invaded cells were analyzed after 24 h. The migration assay was performed as described for the invasion assay without Matrigel coating.

### 4.13. Western Blotting Analysis

Western blotting was performed as described [30,32] using the following antibodies: rabbit polyclonal antibodies against MASTL (Abgent, San Diego, CA, USA); phospho-ENSA (Ser67)/ARPP19 (Ser62), ENSA, cleaved PARP (Asp214), AKT, phospho-Chk2 (Thr68) (Cell Signaling Technology, Danvers, MA, USA); rabbit monoclonal antibody against phospho-c-Myc (Ser 62) (Abcam, Cambridge, UK); mouse monoclonal antibody against caspase-2 (Cell Signaling Technology, Danvers, MA, USA), and c-Myc (Santa Cruz Biotechnology, Inc., Dallas, TX, USA); and a mouse polyclonal antibody against β-actin (Santa Cruz Biotechnology, Inc.).

### 4.14. PP2A Activity Assay

The PP2A activity assay was performed as previously described [30], and the PP2A phosphatase assay according to the manufacturer’s protocol (RediPlate 96 EnzChek serine/threonine phosphatase assay kit; Invitrogen). Okadaic acid (OA) (50 nM; Sigma-Aldrich) or forskolin (40 µM; Sigma-Aldrich) were used for the inhibition or activation of PP2A as negative and positive controls, respectively.

### 4.15. GSEA Analysis

MCF7 cells were treated with DMSO or 250 nM MKI-2 for 24 h. RNA-sequencing was performed at e-Biogen (Seoul, Korea), and the data were analyzed by GSEA software [34].

### 4.16. Oocyte Culture and Treatment

All procedures for mouse care and use were conducted in accordance with the guidelines and approved by the Institutional Animal Care and Use Committees of Sungkyunkwan University. First, 3–4-week-old CD1 female mice were purchased from a local company (Koatech, Pyeongtaek, Korea). To collect fully grown germinal vesicle (GV) stage oocytes, female mice were injected with 5 IU of a pregnant mere’s serum gonadotropin (PMSG). After 48 h, oocytes were collected in M2 medium (M7167) fortified with 0.4% bovine serum albumin (BSA; A7906) and 100 µM of 3-isobuthly-1-methylxanthine (IBMX) to prevent meiotic maturation. During in vitro maturation, oocytes were cultured in IBMX-free M2 medium covered with mineral oil (M5310) at 37 °C in a 5% CO2 atmosphere for 16 h. To collect ovulated oocytes, female mice were induced by intraperitoneal injections of 5 IU of a pregnant mare’s serum gonadotrophin, followed by 5 IU human chorionic gonadotrophin (hCG) after 48–52 h. Mature oocytes were collected 15 h later from the oviduct into the M2 medium and briefly incubated in 300 μg/mL hyaluronidase (H4272) to remove cumulus cells. For chemical treatment, the indicated concentration of chemicals or an equivalent amount of DMSO was added to the culture medium.

### 4.17. Statistical Analysis

The two-tailed Student’s *t*-test was performed to analyze statistical differences between groups. *p*-values of less than 0.05 were considered statistically significant. Excel was employed for statistical analyses.

## 5. Conclusions

Although MASTL is an attractive target for anticancer treatment, MASTL inhibitors with nM range potency and anticancer efficacy have not been reported. The present study newly identified and characterized a second-generation MASTL inhibitor MKI-2 with nM range potency and anticancer efficacy. In addition, we demonstrated the mode of action of MKI-2 targeting of MASTL-PP2A regulation in breast cancer cells. Thus, our data reports a novel MASTL inhibitor for development as a small molecule inhibitor for therapy in MASTL-overexpressed cancers, such as breast cancer.

## Data Availability

The data are available within the article or from the corresponding.
